# CYP2C19 Genetic Variants Associated with Clopidogrel Resistance and Major Adverse Cardiovascular Events in Vietnamese Patients Undergoing Percutaneous Coronary Intervention

**DOI:** 10.2174/011871529X384209250812065418

**Published:** 2025-08-19

**Authors:** Toan Nguyen Duy, Oanh Nguyen Oanh, Kien Nguyen Trung, Khoa Le Ha, Huyen Hoang Thi Kim, Huong Nguyen Thi Lien, Don Dao Van

**Affiliations:** 1 Cardiovascular Center, Military Hospital 103, Vietnam Military Medical University, Hanoi, Vietnam;; 2 Center of Hematology and Blood Transfusion, Military Hospital 103, Vietnam Military Medical University, Hanoi, Vietnam;; 3 Medical Student, Hanoi Medical University, Hanoi, Vietnam;; 4 Department of Clinical Pharmacy, Hanoi University of Pharmacy, Hanoi, Vietnam;; 5 Institute of Pharmacy Training, Vietnam Military Medical University, Hanoi, Vietnam;; 6 Japan Technology Development and Import-Export Single Member Limited Liability Company, Hanoi, Vietnam

**Keywords:** Acute coronary syndrome, cytochrome P-450 CYP2C19, clopidogrel, percutaneous coronary intervention

## Abstract

**Introduction:**

Acute coronary syndrome (ACS) is a leading cause of death, and clopidogrel resistance remains a major challenge in its treatment. This study aims to determine the impact of CYP2C19 genetic variants on clopidogrel resistance (CR) and major adverse cardiovascular events (MACEs) in Vietnamese patients undergoing percutaneous coronary intervention (PCI).

**Methods:**

We carried out a descriptive cross-sectional study, supplemented by a prospective longitudinal follow-up, on 113 ACS patients undergoing PCI with drug-eluting stent implantation at the Department of Cardiology, Military Hospital 103, from January 2015 to May 2018. We excluded patients with a decreased platelet count (< 100 × 10^9/L), a decreased estimated glomerular filtration rate (< 15 mL/min), ongoing bleeding, coagulation disorders, planned or recent surgery, or a coexisting malignancy. CR was defined as platelet aggregation ≥ 46%. The Amplification Refractory Mutation System (ARMS)-PCR was used to determine the CYP2C19 genotype and phenotype. Causes leading to patient readmission, such as angina, recurrent acute myocardial infarction, stroke, or death within 30 days, were recorded as MACEs.

**Results:**

The rate of CR was 29.9% (33/113 patients), and the incidence of MACEs was 15.9% (18/113 patients). The frequencies of CYP2C192 and CYP2C193 polymorphisms, as well as the PM CYP2C19 phenotype, were higher in the CR and MACE groups compared to those without these characteristics (*p* = 0.24, 0.006, and < 0.001, respectively). The PM CYP2C19 phenotype was predictive of 30-day MACEs in ACS patients undergoing PCI with stent implantation (*p* < 0.001).

**Discussion:**

Our findings demonstrate a strong association between CYP2C19 PM phenotypes and increased risks of both clopidogrel resistance and 30-day MACEs in Vietnamese ACS patients undergoing PCI. These results underscore the clinical significance of CYP2C19 genotyping in optimizing antiplatelet therapy and enhancing outcomes in this patient population.

**Conclusion:**

PM CYP2C19 phenotypes were associated with an increased risk of CR and MACEs in Vietnamese patients undergoing PCI with stent implantation.

## 1 INTRODUCTION

Acute coronary syndrome (ACS) includes unstable angina, non-ST-segment elevation myocardial infarction (NSTEMI), and ST-segment elevation myocardial infarction (STEMI) [1]. ACS is the leading cause of cardiovascular death and subsequent serious complications [2, 3]. A study in Europe in 2018 showed that there were 29.4 million people with coronary syndrome, of which 5.5 million were new cases each year. About 1.7 million people die from coronary heart disease annually, accounting for about 20% of total deaths [4]. There are no complete statistics on the incidence and mortality rates of ACS in Vietnam; however, ACS is considered one of the leading causes of in-hospital death. The American Heart Association guidelines recommend the use of dual antiplatelet therapy (a combination of aspirin and clopidogrel) as standard treatment for ACS to reduce major adverse cardiovascular events (MACEs) [5]. However, during treatment, some patients remain resistant to clopidogrel, leading to an increased rate of MACEs, which include recurrent myocardial infarction, stroke, angina pectoris, and cardiovascular death.

Inadequate clopidogrel response, leading to reduced platelet inhibition, also known as CR, is common. Several factors are involved, including genetic factors. Variations in the CYP2C19 gene can impact the effectiveness of clopidogrel, potentially reducing the functional capacity of the CYP2C19 enzyme. Consequently, individuals with loss-of-function (LoF) alleles of CYP2C19 who are prescribed clopidogrel may encounter diminished drug efficacy [6]. The Food and Drug Administration (FDA) issued a boxed warning for clopidogrel, emphasizing that individuals with two reduced-function CYP2C19 alleles (*2 and *3), classified as poor metabolizers, exhibit decreased enzyme activity, which in turn reduces the drug's effectiveness [7]. This gene is not only associated with clopidogrel resistance but also with the frequency of MACEs [8, 9]. Biswas *et al*. reported that patients receiving clopidogrel and possessing one or two CYP2C19 LoF alleles who underwent PCI exhibited a significantly higher risk of MACEs compared to those without these alleles (OR: 1.71, 95% CI: 1.51-1.94, *p* < 0.001) [10].

In Vietnam, patients hospitalized for ACS are often treated with percutaneous coronary intervention (PCI) involving drug-eluting stent implantation and receive intensive dual antiplatelet therapy in accordance with guidelines. However, research on the relationship between CYP2C19 gene variants, clopidogrel resistance (CR), and major adverse cardiovascular events (MACEs) remains limited. In this study, we hypothesized that CYP2C19 gene variants are associated with both CR and MACEs in patients undergoing PCI with stent implantation.

## 2 MATERIALS AND METHODS

### 2.1 Patients

We conducted a descriptive cross-sectional study, supplemented by a prospective longitudinal follow-up, on 113 ACS patients who underwent PCI with drug-eluting stent implantation between January 2015 and May 2018 at the Department of Cardiology, Military Hospital 103, Hanoi, Vietnam.

The sample size was estimated based on the formula:


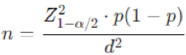


According to a study by Giantini A. *et al*. (2022) [6], the rates of CR and MACEs in patients with ACS were 49.8% and 14.9%, respectively. Substituting into the formula, using a significance level (α) of 0.05 (Z_1-α_^2^ = 1.96) and a margin of error (d) of 10% (d = 0.1), the minimum sample sizes calculated were 96 and 49 patients, respectively. In our study, we used a sample size of 113 patients.

All patients were uniformly treated according to the 2014 ESC/EACTS Guidelines on myocardial revascularization, following a dual antiplatelet regimen. This included a loading dose of 600 mg oral clopidogrel, 324 mg of aspirin, 40 mg of atorvastatin, and 0.5-1 mg of enoxaparin every 12 hours for 3-5 days, followed by maintenance therapy with 75 mg of oral clopidogrel combined with 81 mg of aspirin and 10 mg of atorvastatin. We excluded patients with decreased platelet count < 100 G/l, decreased glomerular filtration rate < 15 ml/min, ongoing bleeding, coagulation disorders, surgical intervention, or accompanying malignancy.

Demographic data, cardiovascular history, and clinical and laboratory characteristics were obtained.

This study was approved by the Institutional Review Board (IRB) of Vietnam Military Medical University, under approval number 1068/2019/VMMU-IRB. All procedures were conducted in accordance with the Declaration of Helsinki. Written informed consent was obtained from all participants prior to their inclusion in the study.

### 2.2 Maximal Platelet Aggregation and Clopidogrel Resistance

The blood sample was taken five days after administering the maintenance dose of clopidogrel. Fasting venous blood was collected using a BD 23G needle into a BD vacuum tube (USA). Four milliliters of fasting peripheral venous blood were centrifuged for 10 minutes at room temperature to isolate the patient's platelet-rich plasma and platelet-poor plasma at speeds of 120G and 850G, respectively. Maximum platelet aggregation (MPA) was evaluated using light transmission aggregometry, with 5 µM ADP used as a platelet aggregation stimulant. The measurement was performed on the Chronolog 560VS analysis system (USA). MPA values higher than 46% were defined as CR [11].

### 2.3 CYP2C19 Gene Polymorphisms

CYP2C19 gene polymorphisms were performed using the Amplification Refractory Mutation System PCR (ARMS-PCR) and Sanger sequencing. Peripheral blood DNA extraction was performed using the Qiagen Mini DNA Extraction Kit, according to the manufacturer's guidelines. The concentration and purity of the extracted DNA samples were assessed using a SpectraMax QuickDrop instrument. The obtained DNA was diluted with deionized water to achieve a concentration of approximately 20 ng/µL, ensuring a purity range of A260/280 between 1.8 and 2.0. It was then stored at -20°C.

The primer pair targeting the CYP2C19 gene sequence with the polymorphism was created using Primer Blast software from NCBI. The sizes of the sequences identifying the *2 and *3 alleles were 203 bp and 159 bp, respectively. The primer pair sequence was as follows:

The mutation was detected through a random selection process using Sanger sequencing, conducted on the SeqStudio Genetic Analyzer, employing the BigDye Terminator v3.1 Cycle Sequencing kit from Thermo Fisher Scientific. Analysis and verification of the ARMS-PCR outcomes were performed using the Bioedit Sequence Alignment Editor.

The classification of clopidogrel-metabolizing CYP2C19 phenotypes based on the identified genotype was as follows:

- Extensive metabolizer CYP2C19 phenotype (EM, characterized by normal CYP2C19 gene): *1/*1 (681GG and 636GG).
- Intermediate metabolizer CYP2C19 phenotype (IM, featuring one loss-of-function CYP2C19 allele): *1/*2 (681GA), *1/*3 (636GA).
- Poor metabolizer CYP2C19 phenotype (PM, carrying two loss-of-function CYP2C19 alleles): *2/*2 (681AA), *2/*3 (681GA and 636GA), *3/*3 (636AA).

### 2.4 Major Adverse Cardiovascular Events

(MACEs) were defined as causes leading to patient readmission, such as angina, recurrent acute myocardial infarction (AMI), stroke, or death within 30 days. These events were confirmed by specialist doctors.

The study diagram is shown in Fig. (**1**).

### 2.5 Statistical Analysis

All continuous normal distribution data were represented by mean and standard deviation and analyzed using the Student t-test or one-way ANOVA test. Categorical data were presented as frequencies with percentages and analyzed using the Chi-square test or Fisher's exact test. Statistical analysis was performed using the Statistical Package for the Social Sciences (SPSS) version 20.0 (Chicago, IL, USA). A *p*-value <0.05 was considered significant.

### 2.6 Inclusion and Exclusion Criteria

The study enrolled 113 patients diagnosed with acute Coronary Syndrome (ACS), who were treated as inpatients and underwent PCI with drug-eluting stent implantation at the Department of Cardiology, Military Hospital 103, between January 2015 and May 2018. Patients were eligible if they were aged ≥18 years and were diagnosed with ACS according to the Third Universal Definition of Myocardial Infarction criteria. All participants had complete clinical and laboratory data required for analysis.

Patients were excluded if they had active bleeding or a history of significant bleeding, had undergone surgery within 7 days prior to admission, had severe renal failure (serum creatinine ≥500 µmol/L), or had a platelet count ≤100 × 10^9^/L. Patients with severe comorbid conditions (such as end-stage malignancy or coma) or those who did not comply with the prescribed treatment regimen were also excluded.

## 3 RESULTS

Table **1** shows that the mean age of the study population was 65.4 ± 11.22 years. Male patients accounted for 77.9%, which was approximately three times higher than the proportion of female patients (22.1%). The smoking rate in the CR group was significantly lower than in the non-CR group (9.1% vs. 31.3%; *p* = 0.013). Of the 113 patients, 18 (15.9%) experienced 30-day MACEs, including 11 patients (9.7%) with angina pectoris and 5 patients (4.4%) with recurrent AMI. Stroke and death each occurred in 0.9% of cases (1 patient in each category). The frequencies of CYP2C19∗2 and CYP2C19∗3 polymorphisms were significantly greater in the CR group than in the non-CR group (*p* = 0.024). Additionally, the prevalence of the PM CYP2C19 phenotype was notably higher in the CR group compared to the non-CR group (*p* = 0.006). The 30-day MACE rate was also significantly elevated in the CR group compared to the non-CR group (10.6% vs. 5.3%; *p* < 0.001).

Table **2** shows that 49 patients (43.4%) had the EM phenotype, 47 patients (40.7%) had the IM phenotype, and 18 patients (15.9%) had the PM phenotype.

Fig. (**2**) shows that the mean MPA value in the EM phenotype was the lowest, while the mean MPA value in the PM phenotype was the highest. The difference was significant with *p* < 0.001.

The multivariate logistic regression model (Table **3**) identified non-smoking status and PM-CYP2C19 phenotypes as independent factors associated with CR. As shown in Table **4**, patients in the MACEs group had higher proportions of females and CR, along with a higher mean MPA value, compared to the non-MACEs group (*p* = 0.026 and *p* < 0.001, respectively). Regarding genetic factors, the *2/*2 and *2/*3 CYP2C19 genotypes, as well as the PM-CYP2C19 phenotype, were significantly more frequent in the MACEs group than in the non-MACEs group (*p* < 0.001). The results of the multivariable Cox regression model in Table **5** show that PM-CYP2C19 phenotype was an independent risk factor for predicting 30-day MACEs in ACS patients (HR=86.542; 95% CI: 18.918 - 395.899; *p* < 0.001).

As the result of Kaplan–Meier analysis in Fig. (**3**), we revealed that patients with PM-CYP2C19 phenotype (red line) exhibited a significantly higher rate of 30-day MACEs than patients without PM-CYP2C19 phenotype (blue line), with a *p*-value of log-rank test < 0.001.

## 4 DISCUSSION

### 4.1 CYP2C19 Genetic Variants in Vietnamese ACS Patients who Underwent Percutaneous Coronary Intervention with Stent Implantation

**Fig. (2):**
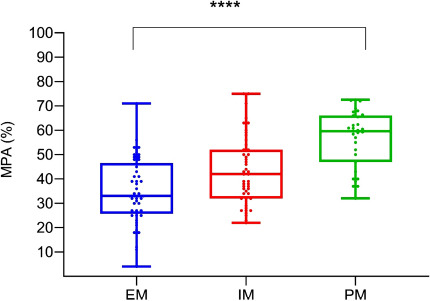
Maximum platelet aggregation according to CYP2C19 phenotypes. (The mean MPA value in the EM phenotype was the lowest, while the mean MPA value in the PM phenotype was the highest. The difference was significant with *p* < 0.001).

CYP2C19 gene testing and antiplatelet treatment with clopidogrel for ACS patients have a specific meaning. The Clinical Pharmacogenetics Implementation Consortium guidelines recommend testing for the CYP2C19 gene when considering clopidogrel for the treatment of ACS [12]. Our results in Table **2** show that 49 patients (43.4%) had the EM phenotype, 47 patients (40.7%) had the IM phenotype, and 18 patients (15.9%) had the PM phenotype. The mean MPA value in the EM phenotype was the lowest, while the mean MPA value in the PM phenotype was the highest. The difference was significant with *p* < 0.001 (Fig. **2**). The distribution of CYP2C19 IM and PM phenotypes in our study was similar to the Korean percutaneous coronary intervention patient group (with IM and PM phenotype rates of 45.8% and 14%) [13] and the Chinese group of patients with acute coronary syndrome (with rates of 45.6% and 14.0%, respectively) [14]. For East Asians in general and Vietnamese people in particular, the proportion of the CYP2C19 IM or PM phenotype is approximately 55%. In contrast, the rate of this phenotype in Europeans, Americans, and Africans is significantly lower (ranging from 25% to 30%) [15]. Thus, we must pay particular attention to the people with IM and PM phenotypes.

In general, platelet aggregation in patients taking clopidogrel increased gradually in subjects: non-carriers of the loss-of-function allele, carriers of one loss-of-function allele, and carriers of two loss-of-function alleles. PM-CYP2C19 phenotype that carries 2 loss-of-function CYP2C19 alleles.

### 4.2 Clopidogrel Resistance and its Association with Genetic and Non-Genetic Factors

During the study period, 113 patients were included, with a mean age of 65.4 ± 11.22 years. Among them, 33 patients (29.2%) had CR, a rate lower than that reported by Giantini A. *et al*. (49.8%) [6] but within the range observed in Asian clinical studies (20–65%) [16]. As shown in Table **1**, the proportion of smokers in the CR group was significantly lower than in the non-CR group (9.1% vs. 31.3%; *p* = 0.013). Additionally, the 30-day MACE rate was significantly higher in the CR group than in the non-CR group (38.2% vs. 7.3%; *p* < 0.001). Similarly, Giantini A. *et al*. reported that smokers had a lower risk of CR compared with non-smokers (OR = 0.37; *p* = 0.001), while CYP2C19 gene hypomethylation increased the risk of CR (OR = 2.13; *p* = 0.037) [6]. The so-called “smoker’s paradox,” observed in several studies, suggests that smoking may confer a protective effect [17, 18]. This phenomenon may be partly explained by the contribution of additional isoenzymes, such as CYP1A2 and CYP2B6, to the metabolism of clopidogrel. Smoking boosts the activity of CYP1A2 (comprising 10% of liver CYP isoenzymes) and CYP2B6, which are crucial in the initial and ultimate phases of clopidogrel metabolism, thereby enhancing the production of its active metabolite [19].

Our results (Table **1**) also indicate that the frequencies of the CYP2C192 and CYP2C193 polymorphisms were significantly higher in the CR group than in the non-CR group (*p* = 0.024). Similarly, the prevalence of the PM CYP2C19 phenotype was significantly greater in the CR group (*p* = 0.006). Similarly, Amin *et al*. reported that CYP2C19*2/*3 polymorphisms were linked to an increased risk of CR compared to the wild-type genotype [20]. The multivariate logistic regression model in Table **3** confirmed that non-smoking status and the PM-CYP2C19 phenotype were independent factors associated with CR in Vietnamese ACS patients.

### 4.3 Major Adverse Cardiovascular Events and their Association with Genetic and Non-genetic Factors

In our study, MACEs were defined as events leading to patient readmission, including angina, recurrent AMI, stroke, or death. Within 30 days, 19 patients experienced MACEs, representing 16.4% of the study population. The proportion of MACEs reported in previous studies varies, likely due to differences in sample size, patient characteristics, and follow-up duration. Giantini A. *et al*. reported a comparable rate, with 30 patients (14.9%) experiencing MACEs, most commonly death (7.5%) [6]. Other studies have identified associations between MACEs and factors such as gender, low eGFR, elevated leukocyte counts [6], comorbidities, and age [21]. However, as shown in Table **2**, we found no significant differences in clinical or laboratory characteristics between the MACE and non-MACE groups, possibly due to the shorter follow-up period in our study.

CYP2C19 gene polymorphism is considered one of the factors that may influence the antiplatelet effect of clopidogrel. In particular, the loss-of-function alleles CYP2C192 and CYP2C193 impair the enzyme activity required for clopidogrel biotransformation, potentially reducing its antiplatelet effectiveness. In intermediate or poor metabolizers (IM/PM), the antiplatelet effect of clopidogrel is diminished, increasing the risk of CR and, consequently, the likelihood of cardiovascular events in patients receiving clopidogrel [22, 23]. Our study found that the proportion of females, the prevalence of CR, and the mean MPA value in the MACEs group were significantly higher than in the non-MACEs group (*p* = 0.026 and *p* < 0.001, respectively). Regarding genetic factors, the proportions of *2/*2 and *2/*3 CYP2C19 genotypes, as well as the PM-CYP2C19 phenotype, were significantly higher in the MACEs group than in the non-MACEs group (*p* < 0.001, Table **4**).

CR reduces platelet aggregation inhibition, causing patients to have a heightened tendency for blood clot formation, which is crucial in the development of MACEs. The response to clopidogrel is influenced by multiple factors, including drug interactions, dosage, adherence, and genetic and epigenetic profiles. Multiple studies have also identified a correlation between CR and an increased risk of MACEs, which is influenced by platelet reactivity. Frere *et al*. reported that individuals with CR had an 8.62-fold greater risk of developing MACEs compared to those who were responsive to the drug (95% CI: 2.31-32.15) [24]. Gurbel *et al*. [11] reported an association between platelet reactivity to ADP, assessed by LTA, and the occurrence of ischemic events (MACEs) within two years after PCI. The MACE group had a significantly higher aggregation percentage than the non-MACE group (46 ± 14% vs. 30 ± 17%, *p* < 0.001 with 5 μM ADP).

Giantini A. *et al*. also suggested a connection between CYP2C19 polymorphisms and MACEs in post-PCI patients over a one-year period [6]. A meta-analysis by Biswas *et al*. [25] found that the presence of the CYP2C19 polymorphism in one allele (either CYP2C19*2 or CYP2C19*3) was associated with an increased risk of MACEs over 12-24 months, with an even higher risk when the polymorphism occurred in both alleles (OR = 2.22, 95% CI = 1.60-3.09). A meta-analysis by Xi *et al*. [26] also reported a higher risk of MACEs in individuals carrying the loss-of-function allele of the CYP2C19 gene over a follow-up period of 6 to 30 months. As the result of Kaplan-Meier analysis in Fig. (**3**), we revealed that patients with PM-CYP2C19 phenotype (red line) exhibited a significantly higher rate of 30-day MACEs than patients without PM-CYP2C19 phenotype (blue line), with a p-value of log-rank test < 0.001. The multivariate Cox regression analysis in Table **5** showed that the PM-CYP2C19 phenotype was an independent risk factor for predicting 30-day MACEs in ACS patients (HR = 86.542; 95% CI: 18.918-395.899; *p* < 0.001). Our results were consistent with those of Giantini A. *et al*. [6]. This study’s results show that mutant carriers of the CYP2C192/3 polymorphism developed MACEs faster than the wild type within one year of observation (HR = 2.12, 95% CI = 1.01-4.46, *p* = 0.047). The CYP2C192 polymorphism results in defective enzyme splicing, whereas the CYP2C193 polymorphism creates a premature stop codon, producing a non-functional protein [27]. These alterations decrease the enzyme's catalytic function, leading to a reduced production of active metabolites, which weakens its platelet aggregation inhibition and increases the risk of MACEs.

This study has several limitations. Firstly, the sample size (n = 113) is modest, and no formal power calculation was performed to determine whether the study was adequately powered to detect the observed associations. Secondly, the follow-up duration was limited to 30 days, which may not have captured all relevant cardiovascular outcomes, particularly for PM carriers whose adverse events might occur later. Thirdly, potential confounding variables, such as medication adherence, lifestyle behaviors (including diet and exercise), and concomitant medications, were not assessed. Finally, the HR for PM-CYP2C19 was markedly high with wide confidence intervals, requiring cautious interpretation. We will conduct a larger sample size and longer follow-up to validate the robustness of these findings, possibly employing bootstrap methods or sensitivity analyses in future studies.

## STUDY LIMITATIONS

This study has several limitations. Firstly, the sample size (n = 113) is modest, and no formal power calculation was performed to determine whether the study was adequately powered to detect the observed associations. Secondly, the follow-up duration was limited to 30 days, which may not capture all relevant cardiovascular outcomes, particularly for PM carriers whose adverse events might occur later. Thirdly, potential confounding variables such as medication adherence, lifestyle behaviors (diet, exercise), and concomitant medications were not assessed. Finally, the HR for PM-CYP2C19 was markedly high with wide confidence intervals, requiring cautious interpretation. We will perform a larger sample size and longer follow-up to validate the robustness of these findings, possibly applying bootstrap methods or sensitivity analyses in further studies.

## CONCLUSION

Gene identification in 113 ACS patients revealed a proportion of patients with EM phenotype of 43.4%, an IM phenotype of 40.7%, and a PM phenotype of 15.9%. The CR rate was 29.9% (33/113 patients). The MACE rate was 16.4% (19/113 patients). The CYP2C19∗2, CYP2C19∗3 polymorphism rates and the PM CYP2C19 genotype rates in the clopidogrel-resistant group or the group with MACEs were higher than those in the group without the above 2 characteristics, *p* =0.24, = *p* =0.006, and < 0.001. The PM-CYP2C19 genotype was predictive of 30-day MACEs in ACS patients undergoing percutaneous coronary intervention with stent implantation, *p* <0.001. Despite limitations, this study highlights the clinical relevance of CYP2C19 genotyping in guiding antiplatelet therapy for populations with a high prevalence of PM alleles. Further studies with larger cohorts and extended follow-up are warranted.

## Figures and Tables

**Fig. (1):**
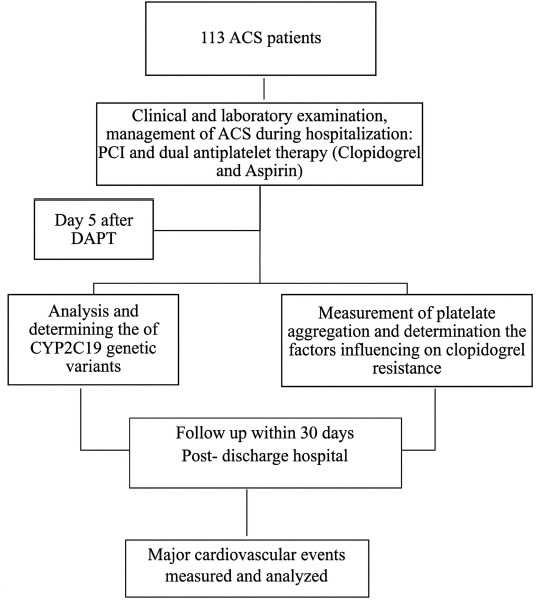
The study diagram.

**Fig. (3):**
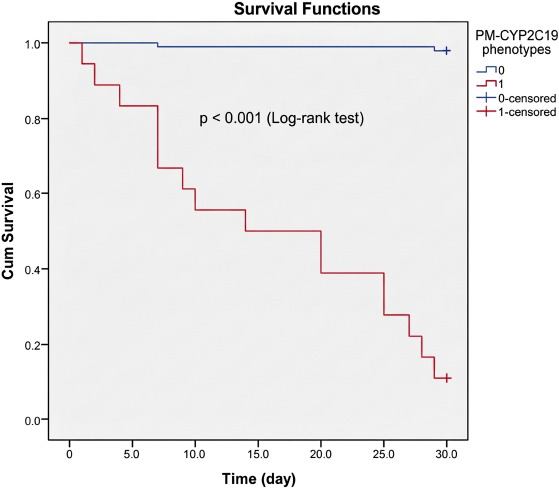
Kaplan-Meier analysis of 30-day MACEs according to CYP2C19-PM phenotype. (The PM-CYP2C19 phenotype (red line) exhibited a significantly higher rate of 30-day MACEs than patients without the PM-CYP2C19 phenotype (blue line), with a *p*-value of log-rank test < 0.001).

**Table 1:** Comparison of demographic, laboratory characteristics, and CYP2C19 genotypes and phenotypes in the CR group and the non-CR group at baseline time.

**Clinical Characteristics and Laboratory Parameters**		**Total (n=113)**	**CR (n=33)**	**Non-CR (n=80)**	***p***
Ages (Years), mean ± SD		65.4 ± 11.22	65.0 ± 11.68	65.56 ± 11.09	0.810
Number of males (n,%)		88 (77.9)	25 (75.8)	63 (78.8)	0.727
BMI (kg/m^2^), mean ± SD		22.48 ± 2.51	22.55 ± 2.92	22.45 ± 2.34	0.871
Smoking (n,%)		28 (24.8)	3 (9.1)	25 (31.3)	0.013
Diabetes (n,%)		25 (22.1)	10 (30.3)	15 (18.8)	0.179
Hypertension (n,%)		70 (61.9)	22 (66.7)	48 (60)	0.507
Lipid disorder (n,%)		74 (65.5)	22 (66.7)	52 (65)	0.865
PLT (G/l), mean ± SD		232.26 ± 74.7	226.28 ± 48.73	234.83 ± 83.61	0.518
STEMI (n,%)		69 (61.1)	13 (39.4)	31 (38.8)	0.949
NSTEMI (n,%)		44 (38.9)	20 (60.6)	49 (61.3)	
LVEF (%), mean ± SD		60.51 ± 11.92	60.64 ± 12.55	60.45 ± 11.72	0.944
β-receptor blockers (n,%)		24 (21.2)	6 (18.2)	18 (22.5)	0.61
ACEI/ARB (n,%)		45 (39.8)	16 (48.5)	29 (36.3)	0.227
PPI (n,%)		39 (34.5)	9 (27.3)	30 (37.5)	0.298
CYP2C19 genotypes	*1/*1	49 (43.4)	8 (24.2)	41 (51.2)	0.024
	*1/*2	40 (35.4)	13 (39.4)	27 (33.8)	
	*1/*3	6 (5.3)	2 (6.1)	4 (5.0)	
	*2/*2	12 (10.6)	6 (18.2)	6 (7.5)	
	*2/*3	5 (4.4)	3 (9.1)	2 (2.5)	
	*3/*3	1 (0.9)	1 (3.0)	0 (0)	
CYP2C19 phenotypes	EM	49 (43.4)	8 (24.2)	41 (51.2)	0.006
	IM	46 (40.7)	15 (45.5)	31 (38.8)	
	PM	18 (15.9)	10 (30.3)	8 (10.0)	
CR (n,%)		33 (29.2)	-	-	-
30-day MACEs (n,%) Angina pectoris Recurrent AMI Stroke Death		18 (15.9) 11 (9.7) 5 (4.4) 1 (0.9) 1 (0.9)	12 (10.6) 6 (5.3) 4 (3.5) 1 (0.9) 1 (0.9)	6 (5.3) 5 (4.4) 1 (0.9) 0 (0) 0 (0)	< 0.001

**Abbreviations:** BMI (Body mass index), PLT (Platelet), eGFR (Estimated glomerular filtration rate), STEMI (ST-elevation myocardial infarction), NSTEMI (Non-ST-elevation myocardial infarction), LVEF (Left Ventricular Ejection Fraction), ACEI (Angiotensin-converting-enzyme inhibitors), ARB (Angiotensin II receptor blocker), PPI (Proton pump inhibitors), CR: clopidogrel resistance; MACEs (major adverse cardiovascular events), AMI (Acute myocardial infarction); EM (Extensive metabolizer), IM (Intermediate metabolizer), PM (Poor metabolizer). Bold value: significant differences.

**Table 2:** Prevalence of CYP2C19 phenotypes and genotypes in ACS patients.

**Phenotypes**	***CYP2C19 Genotypes***	**Number**	**Percentage**	**Overall, n (%)**
EM	*1/*1	49	43.4	49 (43.4)
IM	*1/*2	40	35.4	46 (40.7)
	*1/*3	6	5.3	
PM	*2/*2	12	10.6	18 (15.9)
	*2/*3	5	4.4	
	*3/*3	1	0.9	

**Abbreviations:** EM (Extensive metabolizer), IM (Intermediate metabolizer), PM (Poor metabolizer).

**Table 3:** Multivariate logistic regression analysis on risk factors of CR.

**Variables**	**OR**	**95% CI**	***p*-Value**
Smoking	0.228	0.062-0.839	0.027
PM-CYP2C19 phenotype	3.754	1.279-11.017	0.016

**Abbreviations:** PM (Poor metabolizer). Bold value: significant differences.

**Table 4:** The association between non-genetic factors, Cyp2C19, and 30-day MACEs.

**Factors**		**MACEs (n=18)**	**Non-MACEs (n=95)**	***p***
***Non-genetic Factors***				
Age ≥ 60 (n,%)		12 (66.7)	65 (68.4)	0.884
Females (n,%)		8 (44.4)	17 (17.9)	0.026
BMI ≥ 23 (n,%)		6 (33.3)	38 (40)	0.595
Smoking (n,%)		3 (16.7)	25 (26.3)	0.554
Diabetes (n,%)		7 (38.9)	18 (18.9)	0.117
Hypertension (n,%)		13 (72.2)	57 (60)	0.327
Lipid disorder (n,%)		10 (55.6)	64 (67.4)	0.334
STEMI (n,%)		9 (50)	60 (63.2)	0.294
NSTEMI (n,%)		9 (50)	35 (36.8)	
Number of vessel lesions, mean ± SD		1.11 ± 0.32	1.23 ± 0.47	0.191
1 vessel (n,%)		16 (88.9)	75 (78.9)	0.661
2 vessel (n,%)		2 (11.1)	18 (18.9)	
3 vessels (n,%)		0 (0)	2 (2.1)	
LVEF (%), mean ± SD		60.05 ± 9.27	60.61 ± 12.48	0.865
PLT (G/l), mean ± SD		217.12 ± 46.97	235.25 ± 78.91	0.363
Clopidogrel resistance (n,%)		12 (66.7)	21 (22.1)	< 0.001
MPA *()*, mean ± SD		53.05 ± 12.38	38.73 ± 17.23	< 0.001
eGFR (ml/min), mean ± SD		67.26 ± 20.55	72.0 ± 17.23	0.302
***Genetic Factors***				
CYP2C19 genotypes	*1/*1 (n=50)	1 (5.6)	48 (50.5)	< 0.001
	*1/*2 (n=41)	1 (5.6)	39 (41.1)	0.004
	*1/*3 (n=6)	0 (0)	6 (6.3)	0.587
	*2/*2 (n=13)	11 (61.1)	1 (1.1)	< 0.001
	*2/*3 (n=5)	4 (22.2)	1 (1.1)	0.002
	*3/*3 (n=1)	1 (5.6)	0 (0)	0.159
CYP2C19 phenotypes	EM	1 (5.6)	48 (50.5)	< 0.001
	IM	1 (5.6)	45 (47.4)	0.001
	PM	16 (88.9)	2 (2.1)	< 0.001

**Abbreviations:** BMI (Body mass index), STEMI (ST-elevation myocardial infarction), NSTEMI (Non-ST-elevation myocardial infarction), LVEF (Left Ventricular Ejection Fraction), PLT (Platelet), MPA (Maximal platelet aggregation), eGFR (Estimated glomerular filtration rate), EM (Extensive metabolizer), IM (Intermediate metabolizer), PM (Poor metabolizer). Bold value: significant differences.

**Table 5:** Multivariable cox regression model for predicting 30-day major adverse cardiovascular events.

**Variables**	**HR**	**95% CI**	***p***
Females	2.142	0.715-6.419	0.174
Smoking	0.902	0.168-4.834	0.904
Diabetes	1.416	0.519-3.864	0.497
Hypertension	1.112	0.304-4.073	0.872
PM-CYP2C19 phenotypes	86.542	18.918-395.899	< 0.001

**Abbreviations:** PM (Poor metabolizer). Bold value: significant differences.

## Data Availability

All data generated or analyzed during this study can be obtained from the corresponding author upon reasonable request.

## LIST OF ABBREVIATIONS

ACS: Acute Coronary Syndrome
CR: Clopidogrel Resistance
IRB: Institutional Review Board
MACEs: Major Adverse Cardiovascular Events
MPA: Maximum Platelet Aggregation
STEMI: ST-segment Elevation Myocardial Infarction

## STANDARDS OF REPORTING

STROBE guidelines were followed.
